# A robust approach for industrial small-object detection using an improved faster regional convolutional neural network

**DOI:** 10.1038/s41598-021-02805-y

**Published:** 2021-12-03

**Authors:** Faisal Saeed, Muhammad Jamal Ahmed, Malik Junaid Gul, Kim Jeong Hong, Anand Paul, Muthu Subash Kavitha

**Affiliations:** 1 Kyungpook National University, The School of Computer Science and Engineering, Daegu, 41566 South Korea; 2 Nagasaki University, School of Information and Data Sciences, Nagasaki, Japan

**Keywords:** Mathematics and computing, Computer science

## Abstract

With the increasing pace in the industrial sector, the need for a smart environment is also increasing and the production of industrial products in terms of quality always matters. There is a strong burden on the industrial environment to continue to reduce impulsive downtime, concert deprivation, and safety risks, which needs an efficient solution to detect and improve potential obligations as soon as possible. The systems working in industrial environments for generating industrial products are very fast and generate products rapidly, sometimes leading to faulty products. Therefore, this problem needs to be solved efficiently. Considering this problem in terms of faulty small-object detection, this study proposed an improved faster regional convolutional neural network-based model to detect the faults in the product images. We introduced a novel data-augmentation method along with a bi-cubic interpolation-based feature amplification method. A center loss is also introduced in the loss function to decrease the inter-class similarity issue. The experimental results show that the proposed improved model achieved better classification accuracy for detecting our small faulty objects. The proposed model performs better than the state-of-the-art methods.

## Introduction

Internet of Things (IoTs) plays a key role in a smart world. IoTs are used in many areas as IoTs to manage emergency circumstances or take care of numerous industrial equipments. It is considered that industrial IoTs (IIoTs) have been presaged as a supportive channel of refining functioning competence. The associated IIoT appliances formulate a novel layer that expands the sense of the machinery to control. The active stature of the specific machinery is continuously observed and abreast for their maneuvers. Lately, the extensively used IoT is applied in industrial engineering, ensuing in IIoT. In ordinary IIoT structures, a large number of figures about industrial engineering, typically termed IIoTs, is initially gathered by sensors (detecting terminals), and it is broadcast to the cloud data servers through WSNs or the internet. Subsequently, the progression of industrial production is automatically controlled by the cloud servers, conferring to the gathered IIoT big data^1^. If the cloud servers can figure out the gathered data precisely ahead of the forthcoming anomalous actions, few activities can be reserved ahead to avoid the calamitous demolition. Noticeably, big data analysis and learning algorithms inside the cloud play a significant role in IIoTs to deliver intelligent amenities, such as intelligent transportation and data security^2^. As a significant data science practice, deep learning aims to naturally acquire graded features by assembling numerous conventional artificial neural networks. Illustrative deep learning representations consist of stacked autoencoders, deep belief networks, and deep convolutional neural networks assembled by restricted Boltzmann machines, deep neural networks, and autoencoders, respectively. Currently, deep learning techniques are extensively cast-off in the classification problem, video recovery, and cloud system forecasting.

Currently, the industrial division is developing at an intensifying pace. Thus, it is necessary for each industry to produce products with high quality and without any faults as missing parts in the hardware, e.g., missing screws, untight screws, and missing or misplaced labels. However, high rejection rates of a product exist and arise as a challenging issue for researchers. There can be several reasons for product rejection during quality assurance procedures in the industry. For example, it can be human error^3^ or system misconfiguration. As a result, there is a strong need for an efficient fault detection model. However, this accurate detection system can be inevitable for the industry to produce and distribute good-quality products.

Object detection/identification is an integral part of computer vision. The main difference between the object detection and classification algorithms is that in detection algorithms, bounding boxes are sketched across the object to detect it within the image. Convolutional neural network (CNN)^4–9^ is the traditional method for solving the above problem. CNN is advantageous in learning suitable features from the images; however, the computational efficiency is very low in the traditional CNN model approach. Moreover, a simple CNN model cannot specify the region of interest, where the objects exist; thus, some additional programming logics were used to detect the object region. However, in Faster regional CNN (RCNN), Fast RCNN^10^ and RCNN^11^ are image-detection algorithms for detecting an object in a specific part of the image. The main goal of RCNN is to consider an image and identify the location of the main objects through the region proposals/bounding box in the image via a selective search approach. RCNN finds those bounding boxes by proposing many bounding boxes in the image and examining whether any of them is related to an object. However, the proposals in the RCNN model were designed to apply a selective search approach, which is certainly a slow procedure and causes the bottleneck in the entire process in the system. Thus, Faster RCNN^12^ came into effect in the middle of 2015 to accelerate region proposal processes. The region proposals are based on the image features previously calculated using the normal CNN model.

We created custom data consisting of industrial product images to train the model, where screws and labels exist. In the custom dataset, screws and labels are classified as small objects. Faster RCNN is used as a two-stage deep learning model for detecting these small objects; however, this model has some limitations in detecting small objects. The custom data consist of four types of object class: screw, no-screw, untighten screw, and labels. In each object’s class, there exists a distinct type of object (among our custom classes). Additionally, there is a high imbalance among these diverse objects. These drawbacks of our data are due to spatial distribution along with random frequency and size differences. Therefore, it can make a negative impact on the model during its training. The conventional deep neural networks, such as CNNs, only focus on the object class, which possesses huge data. Thus, it can affect the detection process among the object classes with fewer image samples. The second problem in our dataset is that the objects (screws and labels) are classified into small objects since some screws and labels are very small in size in the images. It is worth noting that the features of such small objects are less than that of the medium or large objects. These less detailed features raise the difficulty of detecting custom objects. Features obtained after the convolution process have semantic information; however, it reduces this detailed information concealed in deep features while performing the pooling process. Although, among our custom data, there is a huge interclass similarity of the object, especially among the screws. Additionally, some factors, such as object type, environmental background, image-shooting angle, and lighting conditions, can increase the object diversity. Thus, it increases the difficulty in detecting the objects.

In this study, we proposed some new techniques to address the above problems. By changing the concept of conventional data-augmentation algorithms, we proposed a data-augmentation method using stitching and oversampling strategies. This method can diminish the negative impact of the class imbalance problem and assemble a new dataset with balanced samples. The pooling process of CNNs reduces the preferential capability of features in individual small objects. Bicubic interpolation is performed for the feature amplification in the last feature map. This interpolation method increases the feature discriminative ability with simple processes. Cross-entropy loss for horizontal and oriented objects is added. Moreover, we introduced a center loss-to-loss function to remove the interclass similarity between our objects. By doing this, our model can classify different objects in the same image. The main contributions of this study are manifolded as follows.

- Fault detection in industrial products (hardware of ATM) using their images is conducted using an enhanced Faster RCNN model.
- We used custom data to train and test the model. The dataset contains ATM hardware images, including screws and labels.
- A new data-augmentation method was introduced based on stitching and oversampling.
- Bicubic interpolation was used to interpolate the feature map in the last layer of VGG16.
- A new center loss was added to the original loss to decrease the interclass similarity between custom objects.
- The enhanced model the Faster RCNN model is tested and compared with DeepBox and EdgeBox techniques. These results are discussed in the result description.

The remainder of the paper is organized as follows. In the background section, we described related work. In the proposed methodology we explained our proposed work in detail and then all experimental work is described in the experimental setup section. In the result section, we discussed our model results in detail finally we concluded our work in the conclusion section.

## Background

Product quality is considered the most important factor for rating the product. Various studies have been conducted to fix the problem of fault identification or missing parts in the manufactured products in the industrial sector. The constant novelties in the IoTs, cyber-physical systems (CPSs), big data, cloud computing, machine learning, and internet of services produced a considerable change in industrial production systems in terms of production rate and quality. Ruppert et al.^13^ presented a detailed overview of the above technologies. They concentrated on the aspects of this organization. It recommends an intelligent space-based layout for the design of Operator 4.0 solutions. Recently, a survey paper was published^14^ to tackle the faults in the industry 4.0-era. Their primary focus was on the fault detection with prediction using ML algorithms. They also discussed some recent machine learning-based techniques as a solution for faults and prediction issues. Marco et al.^15^ conducted a survey on industrial process monitoring (IPM) evaluation. They discussed many evolution trends developed for the betterment of IPM. Their initial focus was on optimizing the IPM detection performance. In^16^, the authors proposed a computer-aided inspection system to monitor the defects in foods, e.g., fruits and vegetables, which are consumed by humans. Their system is based on the feature extraction multilayer neural network of the region of interest; it sorts and grades food products using computer vision techniques. However, the computer-aided inspection system is costly to implement, which is a major drawback. In another research article^17^, a model proposed an automated vision system to detect the flaws of electric motor components since the probability of fault occurrence of defects is more in the manufacturing of electric motor stator due to its manufacturing complexity. Although they used three image-processing techniques to identify the defects, it is not up to the mark of achieving the quality of the overall industrial line. In^18^, the authors introduced the industrial machine vision system and proposed automatic graphical assessment, procedure command, and parts recognition in the robotic industry to recognize or deny objects through image analysis. The fortitude of the improved geometric correspondence among points, execution of basic parameters to represent the points in the Image, and determination of image brightness are needed for better computational methods. Thus, various defect-prevention systems are needed to improve product quality in the industrial sector.

In^19^, the authors proposed a pharmaceutical bottle-packaging-detection system using machine vision technology. They implemented intelligent detection based on machine vision technology to maintain the quality of the pharmaceutical product at a high rate during the pharmaceutical production process. Additionally, they employed a machine vision software called HALCON to control and integrate all hardware parts. In another research article^20^, the authors proposed a tentative machine vision system to control the condition of the color prints of the industrial color printer. They proposed an algorithm for good-quality image acquisition to automate the inspection process of color prints. The major advancements are illumination correction, color dissection, and extraction of features for quality improvement. The authors in the article^21^ presented the performance evaluation technique for pedestrian detection based on Faster RCNN and ACF pedestrian detection. However, RCNN independently finds the areas of the visible object and extracts the feature vectors of the CNN and ACF pedestrian detector. Their results show that their model is much faster than ACF pedestrian detection. Another application of Faster RCNN is proposed in^22^. Here the deep neural network is proposed, along with the integration of a multipath slightly weighted processing chain to incorporate RoI features for improving small vehicle detection in the complicated environment. The backward feature enhancement operation is proposed to deal with large feature maps at lower levels since restricted discriminant information in large feature maps in smaller levels.

## Proposed methodology

**Figure 1:**
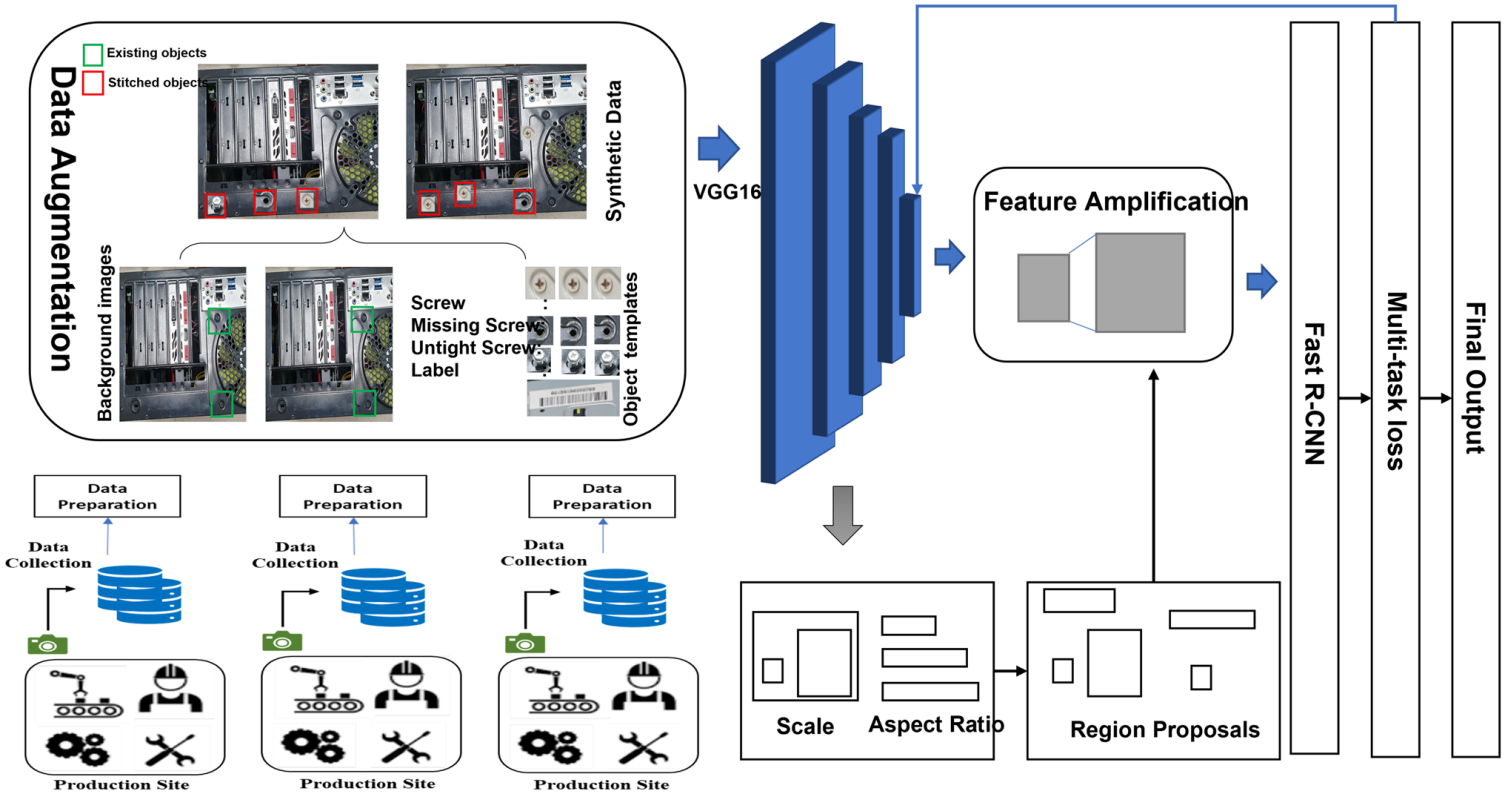
Detailed overview of the proposed architecture.

We proposed an industrial object-detection technique for detecting small objects in the final products, such as screws and labels. Figure 1 shows the complete assembly and structure of the proposed model. From the figure, it can be observed that data are collected from the production site (screws and label images). To perform object detection, we consider Faster RCNN as the subject for research and make enhancements in its performance. Initially, we execute stitching data augmentation and oversampling on collected custom data by developing the frequency of the custom object with a smaller amount of data to produce an advanced dataset. Then, we used VGG16^23^ to extract the features from the input data images. The output feature maps of our VGG16 are very small; feature amplification is conducted to enhance the size of the feature map using bicubic interpolation. In the RPN phase, we assemble multi-shape and multi-scale horizontal anchors. Then, we train the RPN network on the selected negative and positive samples by analyzing and calculating the overlap among ground truths and anchors. In the classification phase, features are amplified for the improved aptitude of feature maps to characterize custom data or objects. Considering the object orientation, we propose a loss function for multitasking, which mutually trains horizontal and oriented bounding boxes and presents the local loss to reduce the inter-class change.

### Data augmentation

**Figure 2:**
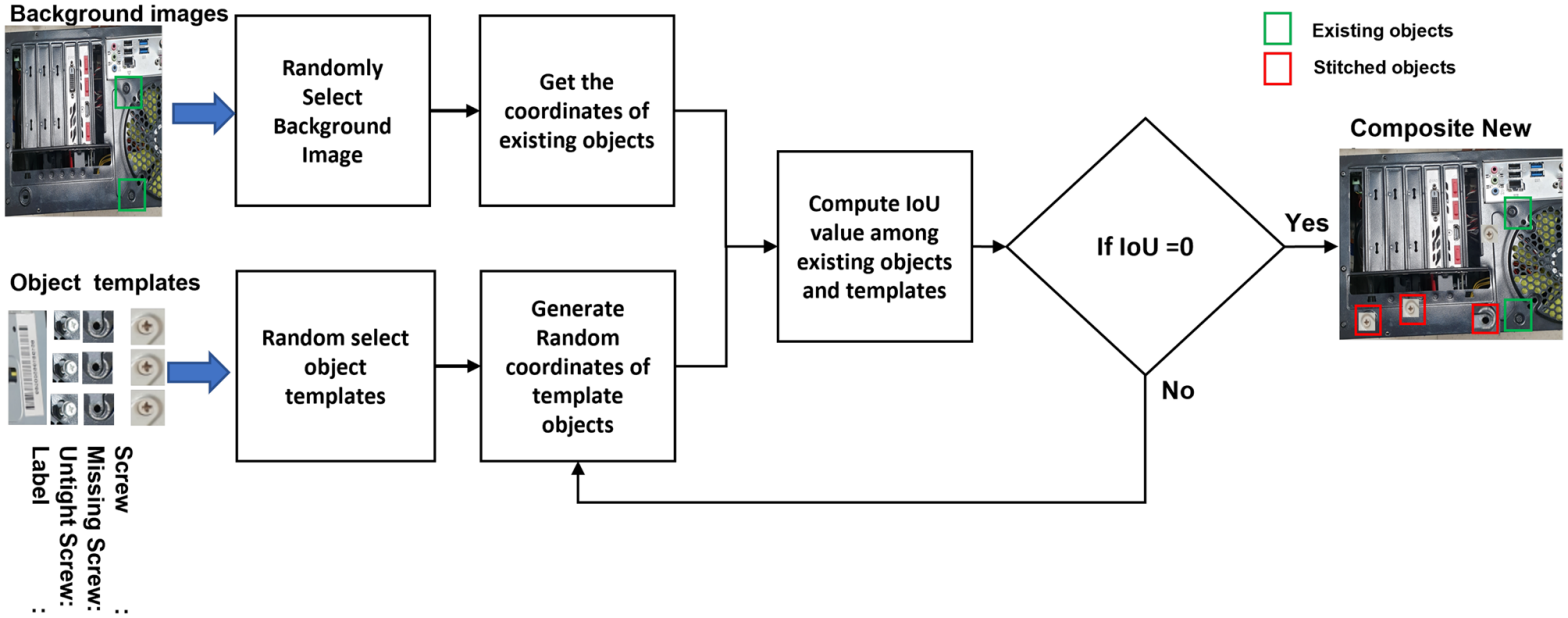
Overall schema of proposed data augmentation method.

The proposed data-augmentation method is expected to counter the class imbalance complications. However, it is a typical and frequent flaw in small-object detection as the location and frequency of diverse objects in custom data are casual. Therefore, it may be under-represented or over presented when expecting a huge variance among diverse objects in the training progression. The factor contributing to the category inequity is the imbalanced class dispersal within a dataset and a batch of samples. Our custom dataset consists of eight types of screws and four types of labels. This shows that a deliberate foreground–foreground class imbalance is present in custom images, which will surely affect the object detection with a slight quantity of data. Additionally, small custom objects take up a smaller amount of image area. The custom object with lesser frequency generally has rarely matched and coordinated anchors, which may intensify our struggle and complication to determine significant material from the network. Bearing in mind that each image consists of backgrounds with a huge image area and only a smaller number of custom objects, this study presents a data-augmentation technique constructed on stitching and oversampling to decline the influence of the imbalance on training evolution as shown in Fig. 2. The primary concept of this procedure is demonstrated as follows:

- Step 1: Training images are rotated at three different angles, i.e., 90ř, 180ř, and 270ř, to create the initial dataset certifying the distinctiveness of object direction.
- Step 2: From the created dataset, segment every custom image of step 1, rendering to the category and position of custom objects to create the object pattern dataset. Since custom objects contain a very small area on the images, the images in the rotated data with less than 10 objects are selected as the dataset for the background image.
- Step 3: In the rotation dataset, sum up the number of custom objects in each class. Then, take numerous types as an extension benchmark. The number of custom objects in each class or category that needs augmentation should be calculated to retain stability and balance among the quantities of custom objects.
- Step 4: To synthesize the new training data or images, we use every object, an arbitrary object from the template dataset, and a specific quantity of images from the background dataset. To condense the imbalanced dispersal of samples within a training batch, we attempt to brand each synthesized image, including all types of objects.
- Step 5: Thinking about the arbitrary position of our objects within the image, arbitrarily create the new locations of small custom objects (screws or labels) in the background images. The overlap between the newly generated and original data are calculated to avoid duplication. The image synthesis process is accomplished when the overlap value becomes 0.
- Step 6: Repeat steps 4 and 5 until the number of custom objects of diverse classes in the dataset becomes balanced.

### Feature amplification

Pooling processes can diminish related features of DNN s; however, there is a possibility of losing some feature maps of small objects. The extension in the deep feature map can be developed through feature amplification, and it can recreate the comprehensive information. In this study, we consider bicubic interpolation to upsurge the features in characterizing small objects with simple procedures. We use VGG16 as a feature extractor. If a custom object with 32 Œ 32 pixels size experiences four pooling operations, the comparable output feature map will be of size 2 Œ 2 pixels. However, this feature map may not completely express the material information of a custom object. The transformations between the presentation and appearances of custom objects of various kinds are comparatively insignificant. However, the comprehensive information of the feature map plays a significant role in differentiating custom objects. Thus, we preferred to operate the feature amplification technique and upsurge the discriminative capability of features for custom objects. For upsampling of the feature map, two foremost approaches are used: interpolation and deconvolution. However, there is a checkboard artifact problem in the deconvolution process, encouraging us not to use it as a comprehensive portrayal of features. Thus, we implement interpolation to expand the image features. We use cubic interpolation to intensify the last feature map. The details of cubic interpolation is given as follows: Assume that the size of the input feature map (A) is m* n, and the size of the target feature map (B) is M*N. Then, as per ratio, we can obtain the coordinates of target feature map B(X,Y) on the input feature map, known as $A(x,y) = A(X^* (m/M),Y^* (n/N))$.

**Figure 3:**
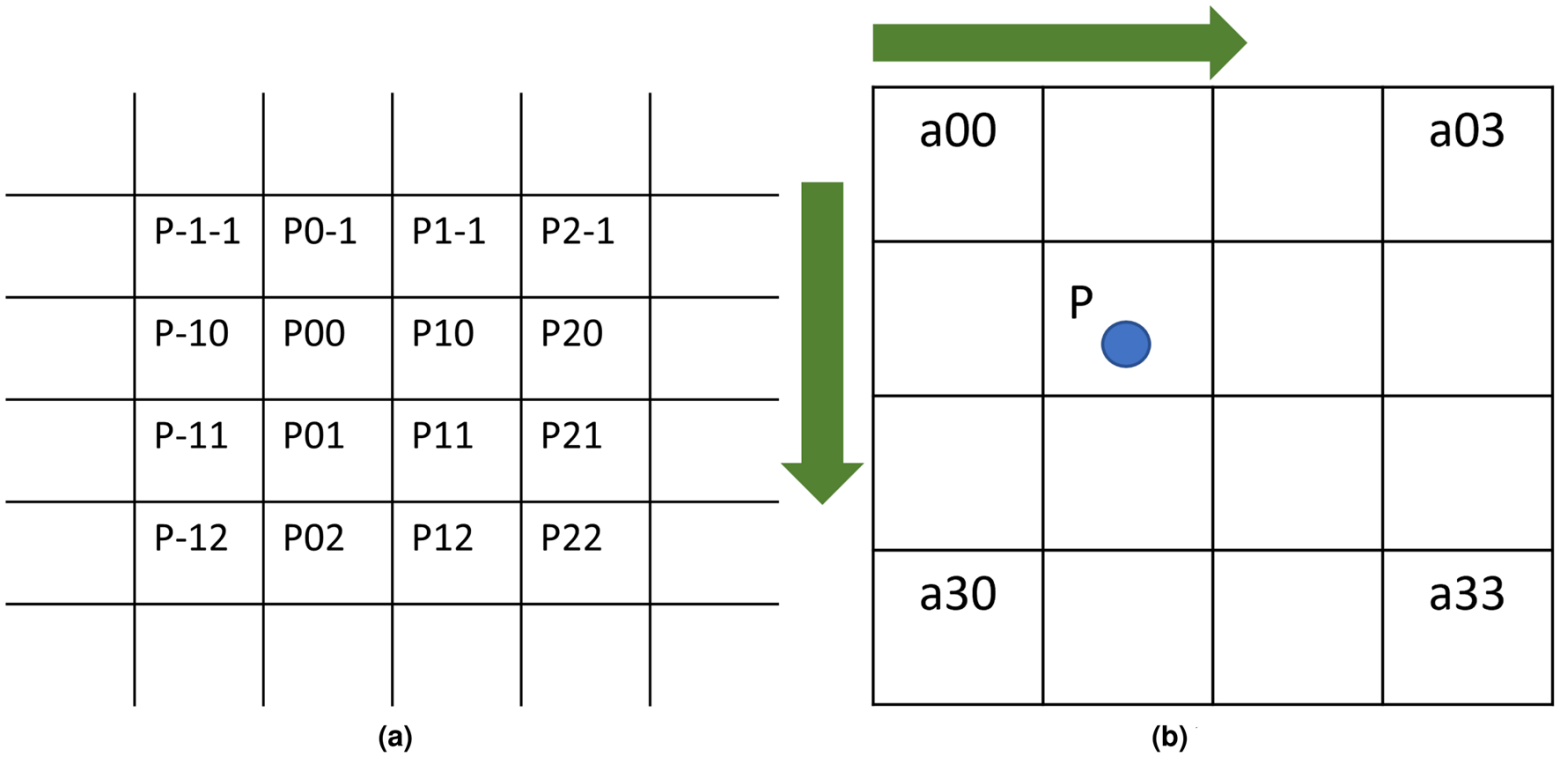
Representation of 16 nearest pixels. (**a**) Representation of 16 nearest Pixel. (**b**) This figure is the illustration of point P on the target feature map.

In the bilinear interpolation method, the last four points of input feature map *A*(*x*, *y*) should be selected; however, the bicubic interpolation method requires 16 nearest pixels as the parameters. These parameters are used for calculating the pixel values of the target feature maps coordinates. This can be seen in Fig. 3a. Figure 3b shows the point P, which is on the place of the target feature map B at the coordinate (X,Y) corresponding to the target feature map. Position coordinates of the point P is like a decimal part. Thus, it is assumed that the coordinate of the point P is $P(x+u,y+v)$. In the coordinate $P(x+u,y+v)$, x and y represent the integer value, whereas u and v represent the decimal part. The primary determination of the bicubic interpolation is to discover the relationship or its coefficient for getting the influence of 16 pixel values at P. The journal function of the bicubic interpolation is shown in Eq. (1).

1 $$\begin{aligned} {\left\{ \begin{array}{ll} (a+2)|x|^{3}-(a+3)|x|^{2}+1  & \quad for \;\; |x|\le 1 \\ a|x|^{3}-5a|x|^{2} +8a|x|-4a  & \quad for \;\; 1 < |x| < 2 \\ 0  & \quad otherwise \end{array}\right. } \end{aligned}$$

### Loss function

The goal of the multitask loss function is to sense oriented and horizontal custom objects (especially the labels) concurrently by merging the loss of oriented bounding boxes with horizontal bounding boxes. Furthermore, the object-detection problem is challenging due to different objects in the dataset and the slight modification of diverse custom objects. Thus, a center loss is proposed in the multi loss function to advance in the discriminative capability of the feature map. Horizontal bounding boxes are used by conventional object-detection approaches to characterize the object location. However, some objects, especially labels, are mostly with random orientation in our case. For an oriented custom object, the location can be defined more precisely by unfolding the coordinates of the four corners. We used two types of anchors^24^ for direction-known object detection. These anchors are called oriented and horizontal anchors. The horizontal anchor mostly contains contextual information of objects, which helps in object recognition. This is the reason the horizontal anchor is relatively preferred to the oriented anchor. Equation (1) shows that the loss function consists of four losses. The first loss is called cross-entropy loss for oriented objects, as shown in Eq. (3). The second loss function is cross-entropy for horizontal objects, as shown in Eq. (4). The third loss is the location regression of the object (particularly labels), as shown in Eq. (5). Finally, the fourth loss is the center loss, as shown in Eq. (6). $\ldots \lambda _1$ and $\lambda _2$ are the balancing parameters.

2 $$\begin{aligned} Loss = L_{cls}^H (p_h,p_h^* ) + L_{cls}^O (p_o,p_o^* ) + \lambda _{1} \sum _{i \in (x,y,w,h)} L_{reg} (t_i,t_i^* ) + \lambda _{2} L_{centerloss} \end{aligned}$$

For classification, $p_h$ is the probability of forecasted horizontal bounding boxes and $p_o$ is the probability of forecasted oriented bounding boxes. Similarly, $p_h^*$ is the true category of horizontal bounding boxes and $p_o^*$ is the true category of oriented bounding boxes.

3 $$\begin{aligned} L_{cls}^H (p_h,p_h^* ) = -\log (p_h) \end{aligned}$$

4 $$\begin{aligned} L_{cls}^O (p_h,p_h^* ) = -\log (p_O) \end{aligned}$$

For location regression, the corners are converted to (x,y,h,w). This is performed to illustrate the position of the oriented and horizontal labels. In Eq. (5), t represents the predicted coordinates, and t* represents the true coordinates.

5 $$\begin{aligned} L_{reg} ={\left\{ \begin{array}{ll}   if \,\, |t-t^* | <  & \quad then \, \, 0.5(t-t^* )^2 \\ otherwise  & \quad |t-t^* |-0.5 \end{array}\right. } \end{aligned}$$

We also include the center loss, as shown in Eq. (6). The purpose of introducing this center loss is to reduce the within-class difference in the features. It also enhances the feature ability in different objects.

6 $$\begin{aligned} L_{center}= 1/2 \sum _{i=1}^n\left\| x_i-c_{y_i} \right\| \end{aligned}$$

Here, the value n is the size of the batch during the classification stage, $c_(y_i )$ represents the center of the feature, and $x_i$ represents the features of the last interpolated feature map.

## Experimental setup

### Dataset description

We used the custom dataset for our model training. First, we captured many industrial product images, such as ATM, computer hardware, and servers. Then, the images are split into four classes: screw, label, missing screw, and untight screw. We captured 917 images. Then, this custom data is augmented using the data-augmentation method. Initially, we had 325, 163, 251, and 178 images for screws, labels, missing screws, and untight screws, respectively. After augmentation, we had 63,013 images. We divided data for training testing and validating. We used 70% of the data as training sets, 20% for testing the model, and 10% for model validation. Table 1 presents the complete description of our dataset.

**Table 1:** Dataset description.

Object type	Original images	After augmentation
Screw	325	19,254
Labels	163	18,754
Missing screw	251	19,478
Untight screw	178	18,527

### Implementation detail

The proposed model is implemented on the custom dataset. The improved Faster RCNN model is implemented in python language using Keras and TensorFlow as basic libraries. The complete model is trained on a machine with the following specifications: Intel Core i5-3570 CPU @ 3.40 GHz 3.80 GHz. Our machine had 64-bit windows operating system with a 64-GB RAM and GPU, called NAVIDIA GeForce GTX1070. The complete specifications are described in Table 2. We used the VGG-16 network pretrained to initialize the RPN and Fast RCNN concurrently. Then, the model was tuned using a custom dataset. During the first training stage, the VGG-16 higher layers and conv3-1 are trained. Previous layers do not need any adjustment since they extract similar features very often. During the second stage, higher layers and the conv5-3 layer in fully connected layers in the Fast RCNN and RPN tuned. We used stochastic gradient descent along with momentum for model training. The momentum and weight decay values were 0.9 and 0.0005, respectively. The primary learning rate was 0.001; however, after every 30,000, 60,000, 60,000, and 30,000 iterations, it decreased by 1/10.

**Table 2:** Detailed environment parameters which are used for implementation.

Sr. no.	Name	Experiment environment parameters
1	Operating system	Windows 10
2	CPU	Intel Core i5-3570 CPU @ 3.40 GHZ 3.80 GHz
3	GPU	NVIDIA GeForce GTX1050
4	Memory	24 GB
5	Development tool	Python 3.6
6	Library	TensorFlow

## Results and discussion

### Comparison based on RoI proposals

**Figure 4:**
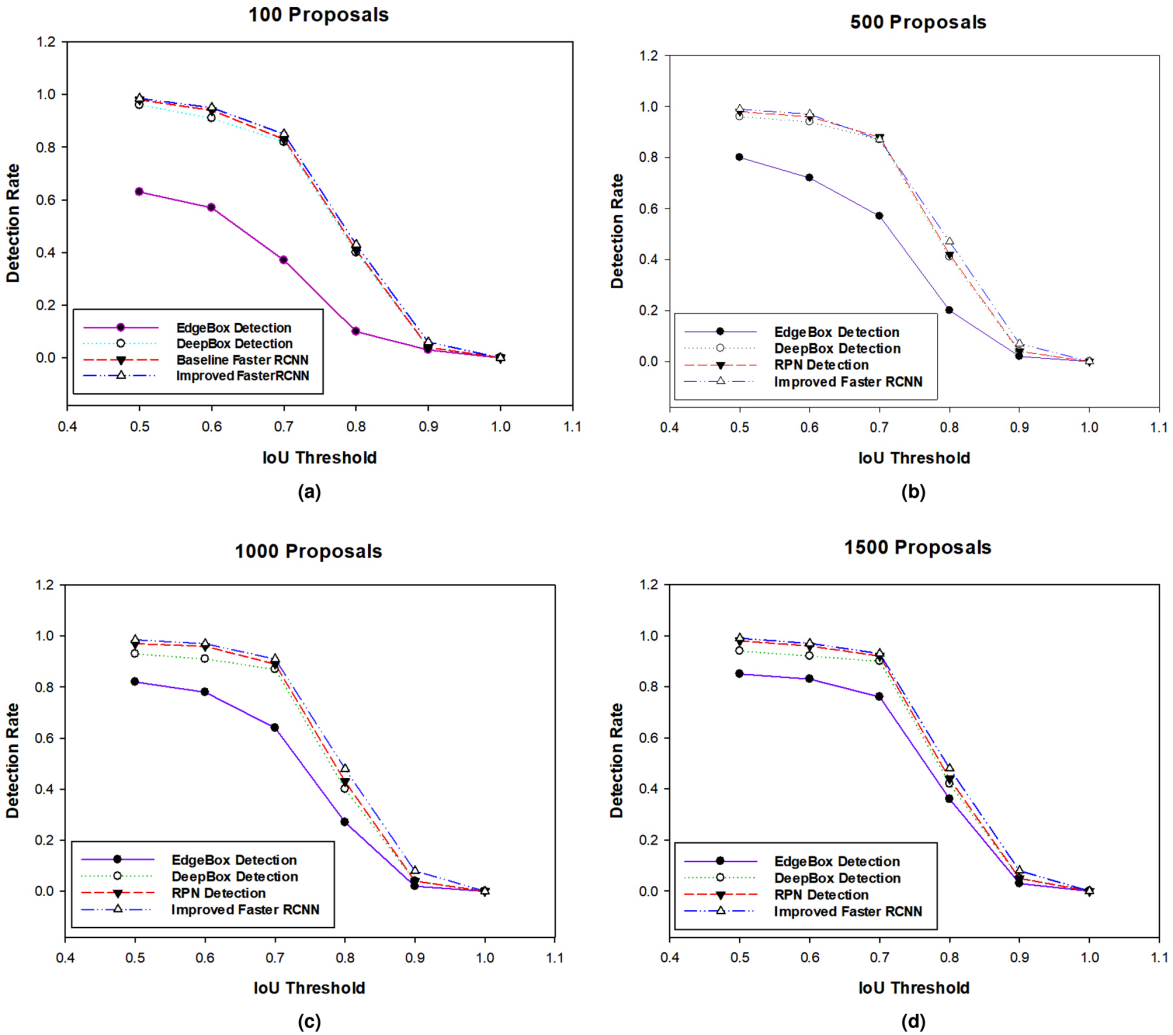
Comparison of improved Faster RCNN based on RoI proposals with baseline Faster RCNN, EdgeBox and DeepBox detection with.

**Figure 5:**
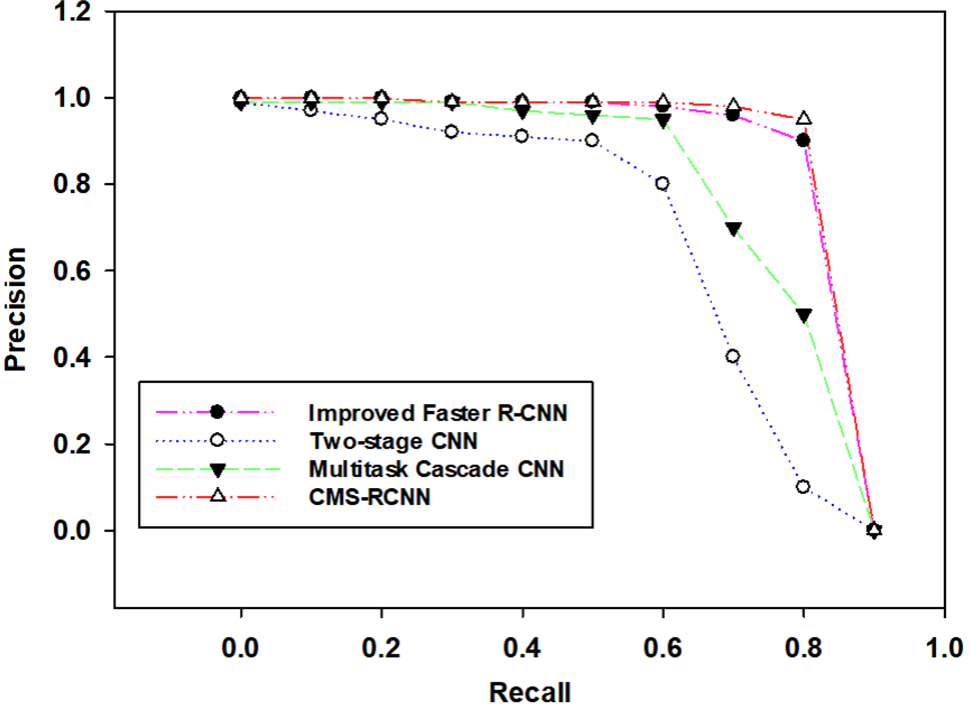
Comparison of the missing part detection module with state-of-the-art methods on our testing dataset.

For the training and testing of our improved Faster RCNN model, we used our custom dataset. The custom dataset consists of four classes: screws, labels, missing screw, and untight screw (Table 1). We trained our model for these classes simultaneously. Initially, training and testing accuracies were calculated during the model training and testing. The comparison of our improved region proposal network with other state-of-the-art methods, such as DeepBox^25^ and EdgeBox^26^, is conducted and described in Fig. 4a–d. EdgeBox evaluates every proposal’s abjectness on the idea of supply edge responses using the sliding window method. However, DeepBox is a method that can rerank object proposals. We retrain the EdgeBox and DeepBox models on our custom-training dataset to calculate the RoI proposals for comparison evaluation. We used^26^ to calculate the detection rate. From Fig. 4, our RPN and DeepBox have relatively better performance. Perhaps, it is not surprising that learning-based methods outperform other heuristic algorithms. RPN uses deep CNNs slightly improved its performance compared to DeepBox. However, the time detection of Faster RCNN is lesser than other methods since it has the property of sharing the convolutional layers of the Fast RCNN detector and RPN region proposal method. We also compared the missing part detection performance of Fast RCNN, Faster RCNN, and RCNN on our dataset. For RCNN and Fast RCNN, we use the top 2000 proposals generated by the EdgeBox model^26^. We fine-tune the RCNN model on the pretrained VGG-16 model using our dataset. Fast and Faster RCNNs are fine-tuned on the VGG16 model using our dataset. As shown in Fig. 5, the Faster RCNN performs better than others. Meanwhile, Faster RCNN also comprises of the Fast RCNN component, but its performance is mostly boosted by the RPN module, which is entirely made of a deeply trained CNN.

#### Comparison of the improved faster RCNN model with other region-based CNN models

**Figure 6:**
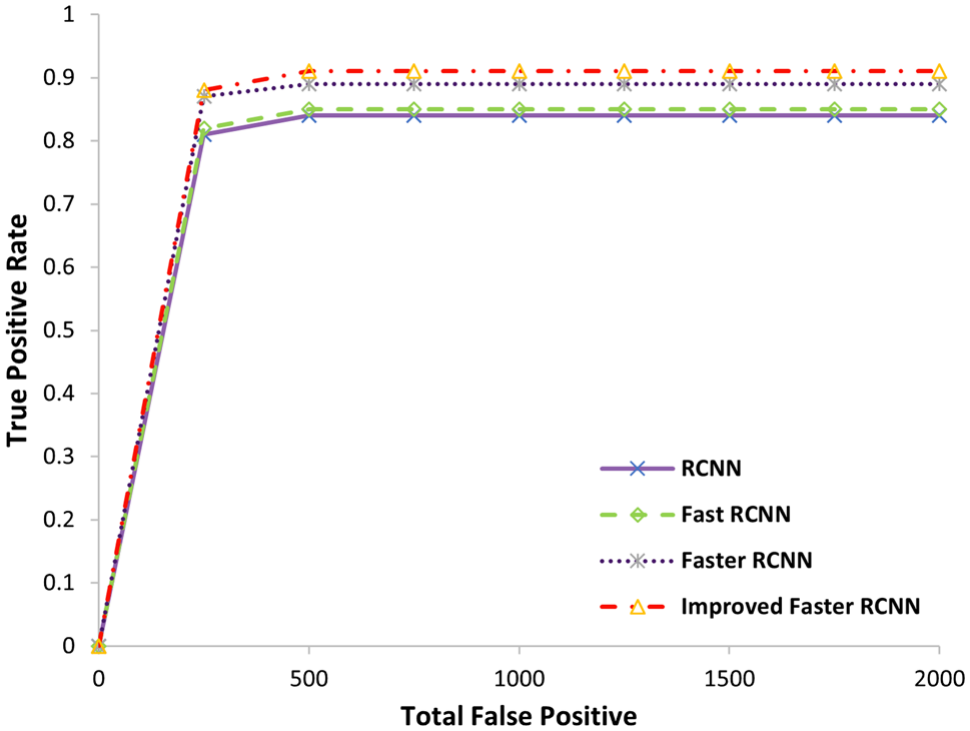
Comparison of faster RCNN with RCNN and Fast RCNN on our used industrial image dataset.

The comparison of Faster RCNN with respect to RCNN and Fast RCNN is also conducted on our dataset. The Fast RCNN and RCNN modules used the best 2000 proposals generated from our experiment, as described in the previous section. We used the pretrained VGG16 model and fine-tuned it to implement RCNN. We employed the method in^27^ to train the CNN model with regression and classification simultaneously to differentiate it from its original implementation^11^. For the Fast RCNN, we also used the same pretrained VGG16 model and fine-tuned it. As shown in Fig. 6, the use of Faster RCNN on industrial images outperforms RCNN and Fast RCNN. The Faster RCNN also contains the Fast RCNN component; most of its performance boost comes from its RPN component centered on a genuinely trained CNN. Similarly, adding new features, such as feature amplification in the RPN module, makes its performance more efficient. Table 3 shows that the improved Faster RCNN runs much quicker than others. From the table it can be observed that the computational time of improved Faster RCNN model is relatively high than baseline model. The reason of high computation time is bicubic interpolation. Moreover, low resolution images such as the image size less than 100*100 gives fast training but very low accuracy which is another limitation of the model.

**Table 3:** Comparison of the proposed Faster RCNN with other region-based modules.

		RCNN	Fast RCNN	Faster RCNN	Improved Faster RCNN
Proposal stage	W.r.t. time	DeepBox: 0.26 s (+ 2.74) = 3.00 s EdgeBox: 2.75 s		0.31 s	0.34 s
’ Refinement stage	CNN input	Images with cropped proposals	Image as input and proposals	Only images as input	Augmented images
	Forward using CNN	Only proposals	1	1	1
	Time	14.5 s	0.25 s	0.07 s	0.6 s
Total	Time	RCNN+DeepBox:- 17.5 s RCNN+EdgeBox:-14.93 s	Fast RCNN+DeepBox:- 3.34 s Fast RCNN+EdgeBox:- 2.92 s	0.39 s	0.42 s

#### Model testing in real-time environment

**Figure 7:**
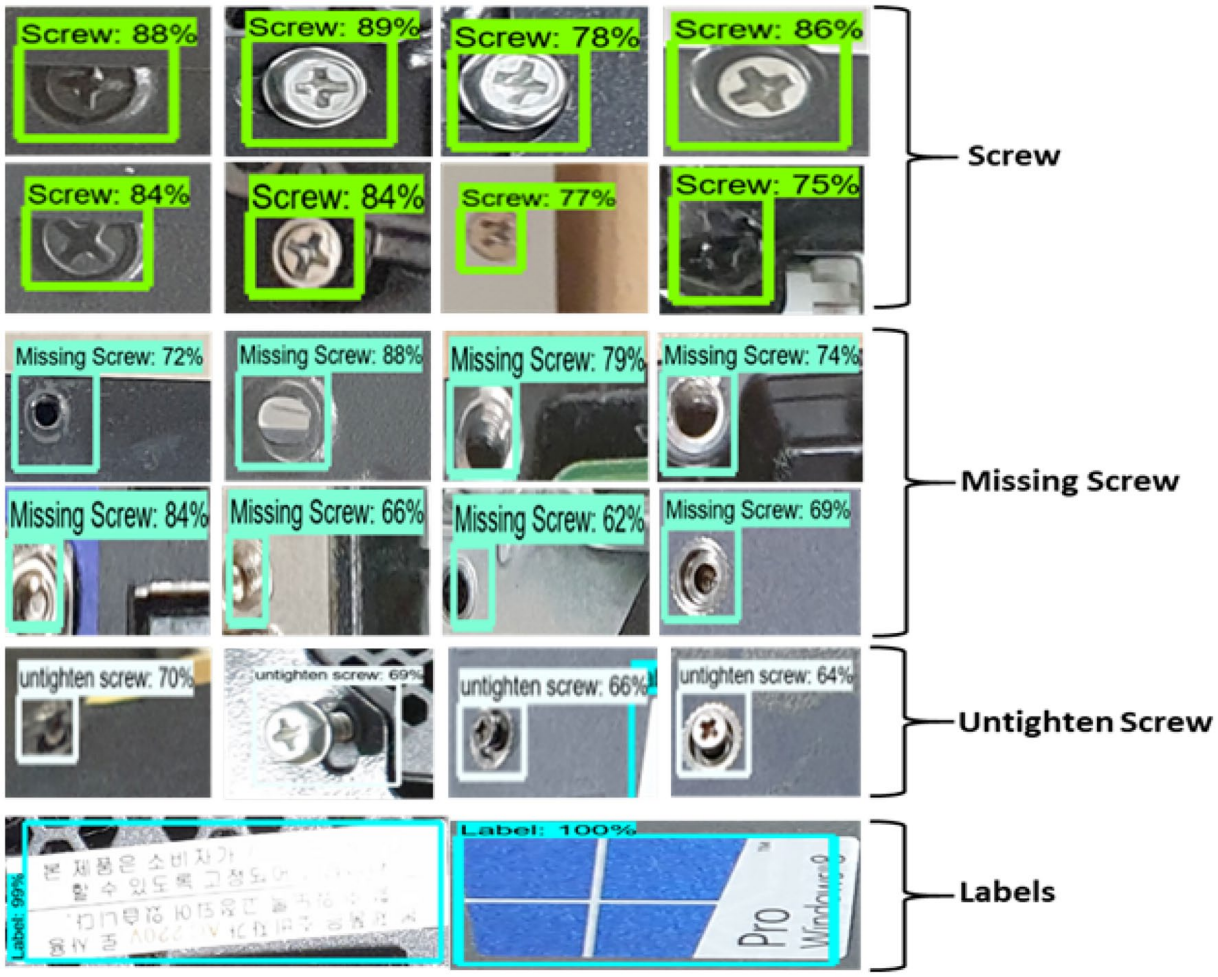
Detection results of the model. The model is tested for four classes, i.e., screws, missing screws, untighten screw, and labels.

We tested our complete proposed Faster RCNN model on our generated testing data and in a real-time environment. We captured images of different products and sent them for testing. These images were then tested one by one. Figure 7 shows the tested results of the model on our defined classes. The rectangles on the images are the region proposals selected by RPN and classified by Fast RCNN.

**Figure 8:**
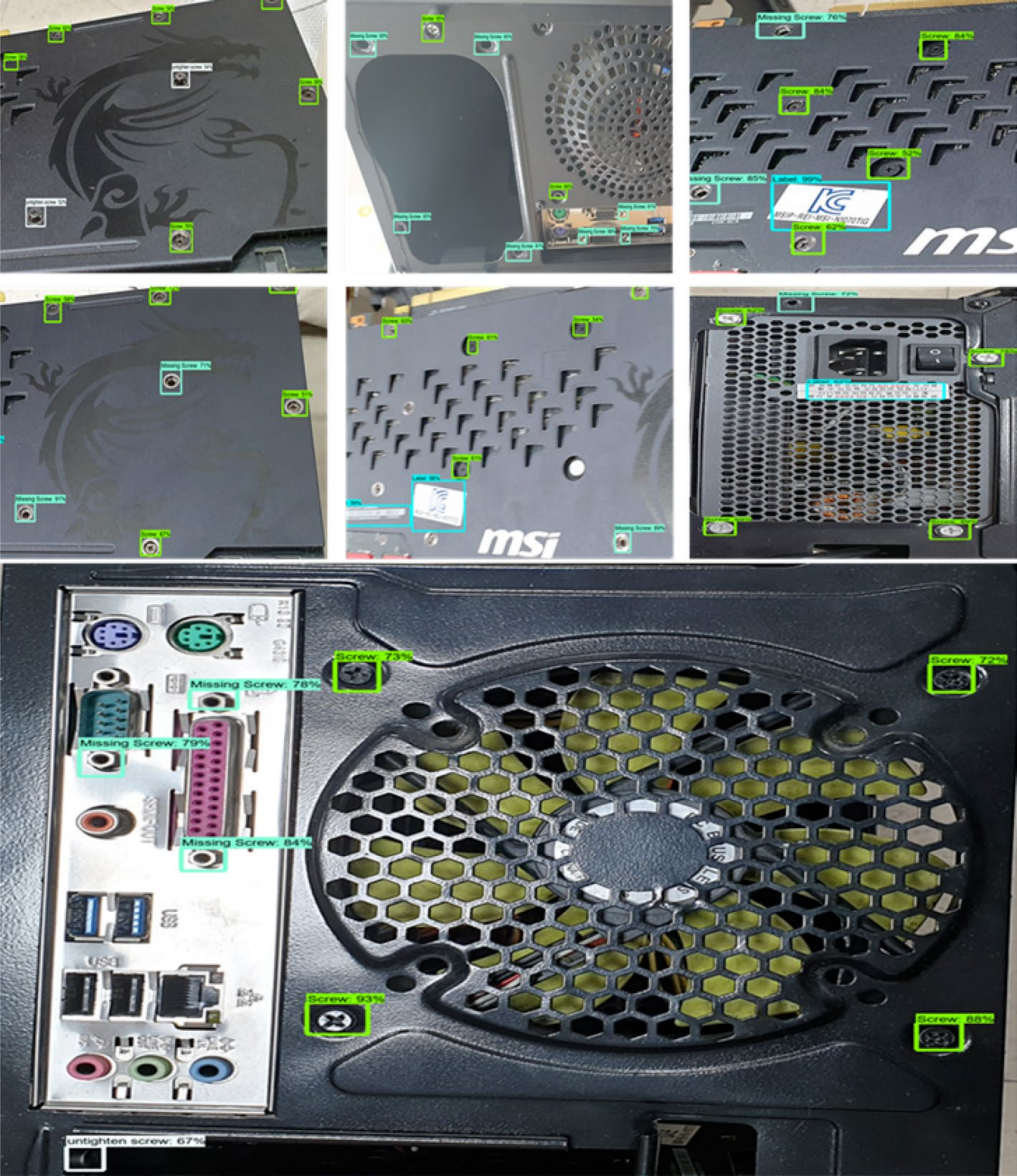
Real-time environmental testing. Model tested on product images, where our four object classes exist, i.e., screws, labels, missing screws, and untight screws.

**Figure 9:**
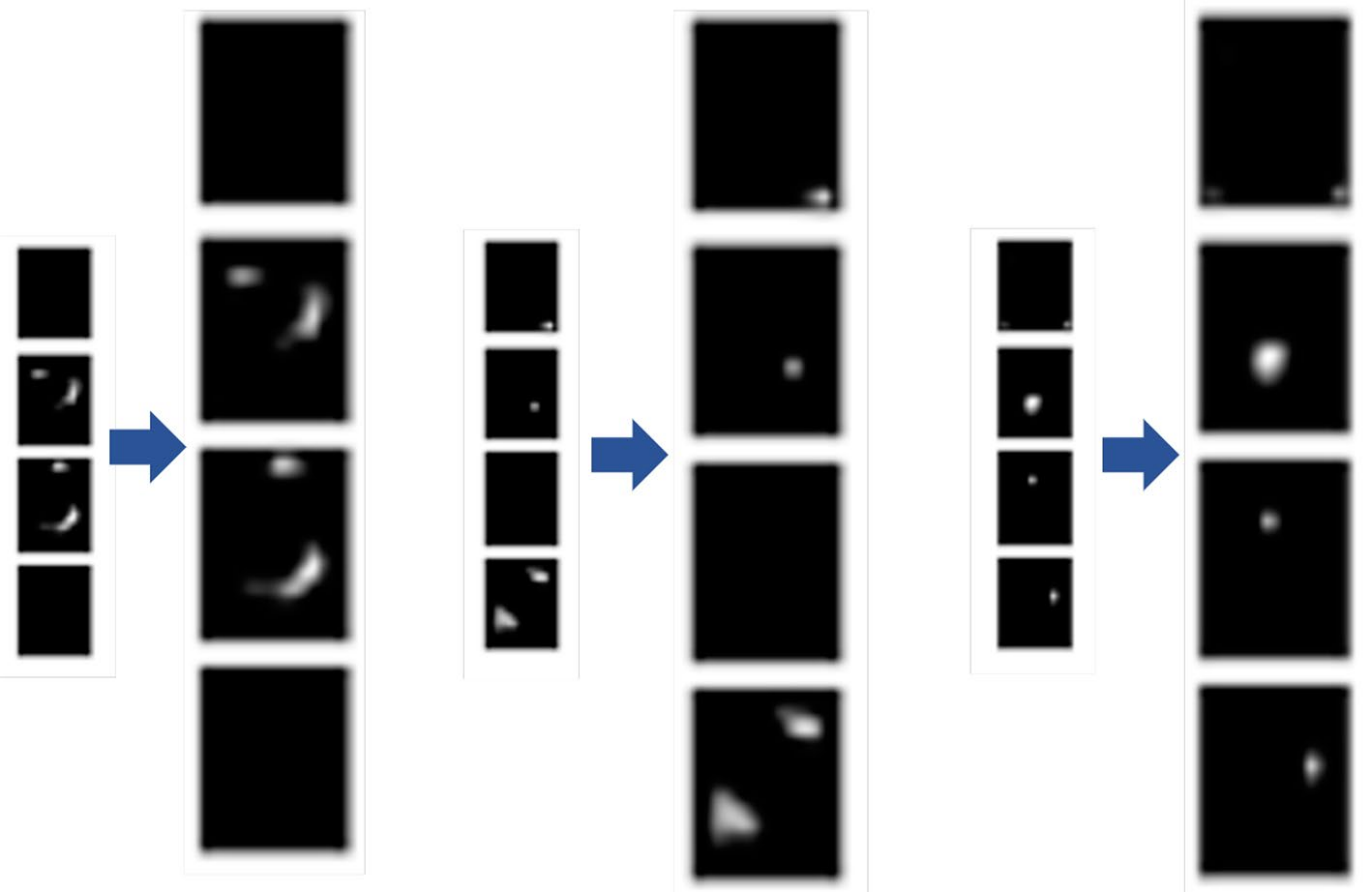
Visualization of the amplified feature map. Amplification is performed by bicubic interpolation.

Figure 8 shows the real-time environmental test results. As shown in the figure, the model detects the diverse type of custom objects. Additionally, our model can efficiently detect the bounding object boxes during determining certain categories of objects. Table 4 presents the results of baseline small-object detection approaches. These methods are tested on our merged custom data, i.e., after augmentation. The overall detection results are better than these baseline approaches since our model is improved in terms of horizontal boxes. The interpolated feature maps can enhance the discriminative capability of features by reestablishing the feature map data. Center loss can increase the gap of different types of objects’ feature maps by reducing the interclass similarities in the features corresponding to the same object type. As a result, the proposed model detection efficiency is relatively higher than others. For FPN^28^ and DFPN^29^, the table shows that DFPN has better accuracy than FPN. DFPN has the functionality of using multilayer features to construct a stronger feature pyramid among diverse feature layers. This functionality enables the model to modify multiscale objects. This is the reason why DFPN has better accuracy than FPN. To prove the effectiveness of the bicubic interpolation on the proposed model, we used our custom dataset to deliberate the enlargement operation of feature maps. Initially, we visualize the enlarged feature map using the bicubic interpolation shown in Fig. 9. To demonstrate the better performance of the bicubic interpolation, we used three interpolation procedures: bicubic interpolation, bilinear interpolation, and nearest-neighbor interpolation for comparison shown in Table 5. Table 5 shows the optimal average precision of the model of three different interpolation methods. Furthermore, the detection rate of the proposed model without enlarging the feature in the last maps is 53.3%, which is less than after performing feature amplification. These tabular results show that bicubic interpolation outperforms the other interpolation methods. Therefore, bicubic interpolation performs better than bilinear 2.0 and 2.5 multiples. Similarly, the nearest-neighbor interpolation 2.0 performance is lesser than bicubic.

**Table 4:** Comparison of proposed model accuracy with other state-of-the-art methods.

Objects	FPN	DFPN	Faster RCNN	Improved Faster RCNN
Screw	83.5	84.3	80.8	89.3
Missing Screw	84.3	79.5	77.5	96.5
Untight Screw	80.8	82.2	82.2	90.2
Label	86.7	81.3	80.3	86.3

**Table 5:** Comparison of Interpolation methods on proposed model accuracy.

Objects	NN 2.0	Bilinear 2.0	Bilinear 2.5	Bicubic interpolation
Screw	66.5	72.1	80.5	89.3
Labels	71.2	71.5	77.5	96.5
Missing Screw	65.7	69.2	75.2	90.2
Untight Screw	55.1	62.3	72.3	86.3

**Figure 10:**
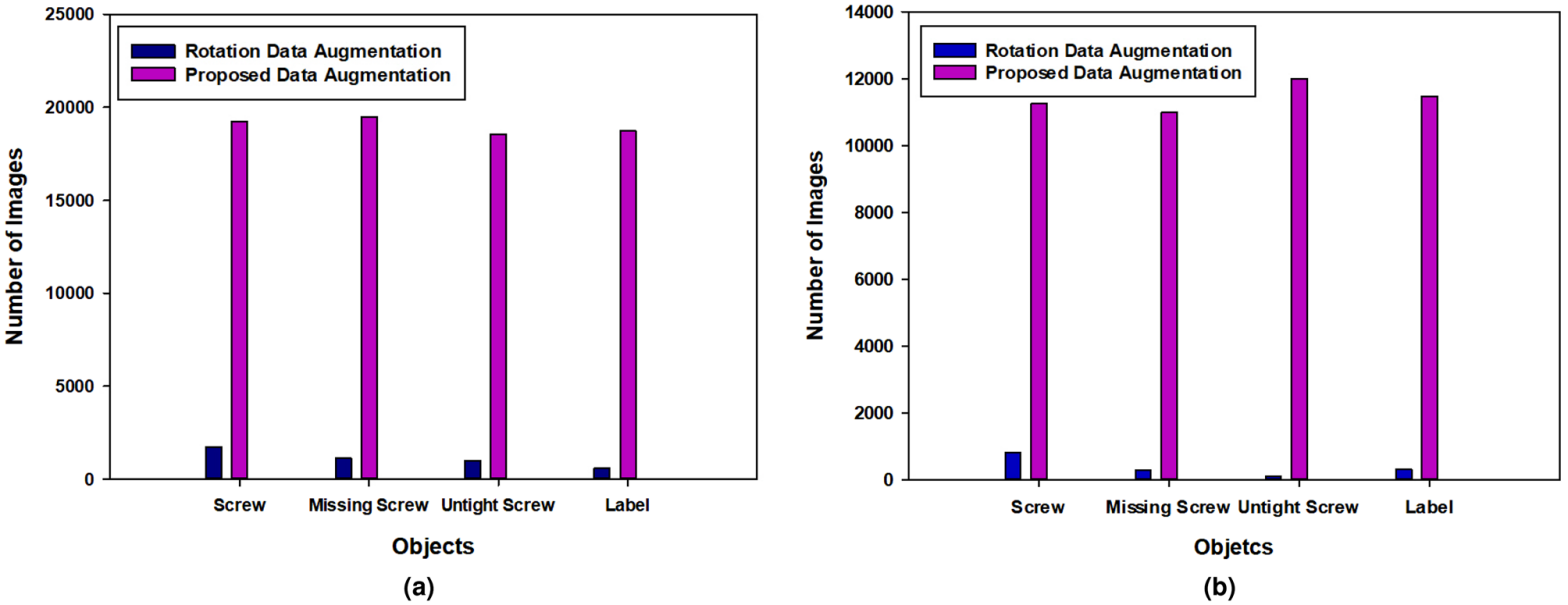
Comparison of the proposed data-augmentation method with rotation-augmentation technique. Left graph a shows the comparison results of the first trial, and right b graph show the comparison results of the second trial.

As described in “Introduction” section, the category of imbalance problem among our custom objects can negatively impact our network’s training. Thus, to remove this impact, we proposed a new data-augmentation technique. Initially, we processed rotation augmentation on our data to enhance the angled variety of our custom objects. As shown in Fig. 10a,b, after rotation augmentation, there is a huge class imbalance among objects ranging from 790 to 1725 in the first trial and 90 to 820 in the second trial. Thus, the rotation-augmentation method could not remove this class imbalance problem even though it increases the difference between objects. From the figures it can be observed that the number of images in each class is almost the same after processing the proposed data-augmentation method on our custom objects. After the first trial, the number of images varies from 18,527 to 19,478 and it varies from 11,000 to 12,100 in the second trial.

## Conclusion

Fault detection in the images is a challenging task, especially in terms of small-object detection. This study proposed an efficient method for solving a challenging problem of industries, i.e., sometimes industrial products, such as spare parts of ATMs or computer hardware, have missing screws and labels, leading to faulty products. Initially, we put this problem as a small-object-detection problem since our custom objects are very small. We used Faster RCNN to detect these small objects. We experienced three problems, class imbalance, fewer features in the last convolutional layers and inter-class similarity using a new data-augmentation method to balance the objects, bicubic interpolation for feature amplification and adding a center loss to multi-loss function to remove inter-class similarity. Our generated data were used for the model. First, the data-augmentation method was processed. Then, the data were sent to the pretrained VGG16 model for feature extraction. Furthermore, bicubic interpolation enlarged these extracted features for RPN. Additionally, Fast RCNN was used for object classification. The comparison of the proposed model with other state-of-the-art methods shows that the proposed model performs better than others. The overall results shows the model performs better comparatively.
